# Bone Mineral Density Changes during Weight Regain following Weight Loss with and without Exercise

**DOI:** 10.3390/nu13082848

**Published:** 2021-08-19

**Authors:** Monica C. Serra, Alice S. Ryan

**Affiliations:** 1 Division of Geriatrics, Gerontology & Palliative Medicine and the Sam & Ann Barshop Institute for Longevity & Aging Studies, Department of Medicine, UT Health San Antonio, San Antonio, TX 78229, USA; 2 San Antonio Geriatric Research Education and Clinical Center (GRECC), South Texas Veterans Health Care System, San Antonio, TX 78229, USA; 3 Division of Gerontology and Palliative Medicine, Department of Medicine, University of Maryland School of Medicine, Baltimore, MD 21201, USA; aryan@som.umaryland.edu; 4 Baltimore GRECC, VA Maryland Health Care System, Baltimore, MD 21201, USA

**Keywords:** bone mineral density, exercise, weight loss, weight regain, aging, postmenopausal women

## Abstract

The purpose of this study was to compare changes in bone mineral density (BMD) over a 6 month follow up (period of weight regain) in overweight, postmenopausal women having previously completed a 6 month weight loss (WL) intervention with and without aerobic exercise (AEX). Women (BMI > 25 kg/m^2^) underwent VO_2_max and DEXA scans at baseline, after 6 months of WL or AEX + WL, and at 12 months ad libitum follow up. Both groups lost ~9% body weight from 0 to 6 months and regained ~2% from 6 to 12 months, while losing ~4% of appendicular lean mass (ALM) across the 12-month study duration. VO_2_max increased 10% from 0 to 6 months and declined 12% from 6 to 12 months for AEX + WL, with no changes for WL. Total body (*p* < 0.01) and total femur (*p* = 0.03) BMD decreased similar between groups across time (combined groups: 0–6 months: total body: −1.2% and total femur: −1.2%; 6–12 months: total body: −0.26% and total femur: −0.09%). Less ALM loss and greater VO_2_max increases during the WL phase were associated with attenuated BMD loss at various anatomical sites during periods of weight regain (6–12 months) *p*’s < 0.05). Results suggest that BMD loss may continue following WL, despite weight regain. Further, this study adds to the literature by suggesting that preventing declines in muscle quality and function during WL may attenuate the loss of BMD during weight regain. Future studies are needed to identify mechanisms underlying WL-induced bone loss so that effective practices can be designed to minimize the loss of BMD during WL and weight maintenance in older women.

## 1. Introduction

Approximately 43% of adults over the age of 60 years are obese [1]. Obesity is associated with a high prevalence of chronic conditions, including heart disease, stroke, type 2 diabetes mellitus (T2DM) and certain types of cancer [2]. Despite the well-documented consequences of excess adiposity in older adults, weight loss (WL) remains controversial in older adults due to concomitant loss of bone mineral density (BMD) [3], especially considering that more than half of the lost weight during weight loss studies is regained within two years [4] so the risk for obesity remains high. Studies suggest that BMD lost during WL is not recovered with weight regain [5,6,7,8,9,10,11], and that bone loss may actually continue at an accelerated rate after weight loss [10,11]. Thus, strategies to promote BMD maintenance and recovery during periods of weight loss and regain, respectively, are needed.

Weight-bearing aerobic exercise (AEX), such as walking and jogging, in weight-stable adults is documented to provide positive effects on BMD, although the results appear to be dependent upon the anatomical region of analyses and degree of skeletal loading [12]. We [13,14] and others [15,16,17,18,19,20,21] have shown that the addition of AEX (alone and when included in multimodal exercise interventions) during WL is beneficial to preserve lean mass and BMD. Though we have shown that participating in AEX during WL did not prevent weight regain or declines in aerobic capacity (VO_2_max) during a 6 month follow-up period [22], little is known about how the addition of AEX during WL affects BMD during periods of weight regain, which we aim to explore in the present analyses. We hypothesize that the women performing AEX during WL will have less BMD loss over a 6 month follow up (period of weight regain) than those who initially participate in WL alone. We also examine whether body composition and fitness factors influenced by WL and AEX (i.e., body weight, lean mass, and VO_2_max) are associated with the change in BMD during the period of weight regain.

## 2. Materials and Methods

### 2.1. Study Design

This randomized controlled trial examined the effects of adding AEX to 6 months of intentional WL, compared to WL alone, on body composition, glucose metabolism, and CVD risk, and how these outcomes were affected after a 6 month follow up (12 month). We have previously published detailed study procedures and the 12 month follow-up results of this study [22]; however, 12 month follow-up effects on BMD were not previously reported. In brief, subjects met with a Registered Dietitian (RD) weekly for 6–8 weeks to learn a weight-stable, heart-healthy diet (daily consumption less than 30% of calories as total fat, 10% as saturated fat, 300 mg of cholesterol, and 2400 mg of sodium). Following dietary stabilization, subjects completed baseline testing (described below). Subjects then entered the 6 month WL phase, which involved meeting weekly with the RD to learn techniques for consuming a hypocaloric (250–350 kcal/d deficit), heart-healthy diet. Half the women were also asked to perform progressive, supervised aerobic treadmill brisk walking/jogging exercise (AEX + WL group) at >85% heart rate reserve for 45 min (plus 5–10 min warm-up and cool-down), three days per week, at our facility. Following the 6 month WL phase of WL or AEX + WL, subjects entered a 6 month follow-up phase (weight regain phase), which provided all women with the option to attend once-monthly (~60 min each) nutrition refresher classes. The AEX + WL group could also continue to use the exercise facility three times weekly for up to 60 min, if desired. We have previously reported attendance during the WL phase (both WL and AEX + WL) to be >85% of available diet and exercise sessions and that during the follow-up (weight regain) phase, women attended 27% of the available exercise sessions (AEX + WL group only) and 29% of available nutrition refresher classes [22]. Clinical Trial Registration: URL: https://clinicaltrials.gov (accessed on 10 March 2021); Unique Identifier: NCT00882141.

### 2.2. Study Subjects

Overweight and obese (BMI >25 kg/m^2^), postmenopausal (age 45–80 years) women were recruited from the Baltimore/Washington area. All women signed University of Maryland Institutional Review Board-approved informed consent forms. Eligible women were sedentary, non-smokers, and weight stable (<2 kg weight change over past year). A medical history, physical examination, resting 12-lead electrocardiogram, and fasting blood profile were performed to exclude those with unstable medical conditions. Subjects with evidence of unstable hypertension, hypertriglyceridemia, heart disease, cancer, liver, renal or hematological disease, orthopedic limitations, or medical conditions deemed to impact participation were excluded.

Fifty-four women, from a larger subset of individuals from a previously published paper (*N* = 174) [23], were selected for analysis based upon complete BMD data at 12 months. In addition to those that dropped out or were excluded due to non-compliance to the interventions (data previously reported [22]), an additional 11 women were excluded due to incomplete BMD data, leaving a final sample of *N* = 26 who completed WL and *N* = 28 who completed AEX + WL at the 12 month follow up.

### 2.3. Procedures

Study procedures listed below occurred at baseline, after 6 months of WL or AEX + WL, and after a 6 month follow up (12 month).

#### 2.3.1. Body Composition and BMD

Height and body weight were measured using a stadiometer and digital scale to calculate body mass index (BMI: weight [kg]/height [m^2^]). Total body and hip and spine regional dual energy x-ray absorptiometry scans (DXA; DPX-IQ; LUNAR Radiation Corp., Madison, WI, USA) were performed to determine BMD (g/cm^2^) of the total body, femoral neck, total femur, greater trochanter, Ward’s triangle, and lumbar spine (L1-L4). T-score for the femoral neck, total femur, and lumbar spine were also calculated. T-scores of −1.0 and above were considered normal, between −1.0 and −2.5 were considered osteopenic, and −2.5 or lower were considered osteoporotic. The 12 month BMD data are new to this analysis. The total body scan was also used to calculate appendicular lean mass (kg).

#### 2.3.2. Cardiorespiratory Fitness

VO_2_max was measured by indirect calorimetry during a graded exercise test on a treadmill as previously described [23]. A test was determined maximal if two of the following criteria were met: respiratory exchange ratio ≥1.0, maximum heart rate >90% of age-predicted maximum (220-age), or a plateau in VO_2_.

### 2.4. Statistical Analyses

Statistics were only run on subjects who completed baseline, 6 and 12 month assessments. A two-way analysis of variance was used to determine if there were group effects, time effects, or groups x time interactions in BMD. The Bonferroni post hoc test was used when the overall effects were significant. Multiple regressions were used to assess relationships between key variables after controlling for age and race. Statistical significance was set at a two-tailed *p* < 0.05. Results are expressed as the mean ± SD for continuous variables and percentage for categorical variables.

## 3. Results

### 3.1. Participant Characteristics

Subject characteristics may be viewed in Table 1. Overall, 40% of women were African American and 60% Caucasian. At baseline, groups were similar with regard to mean body weight, BMI, and BMD; however, the WL group was slightly older (~4 years; *p* = 0.02) than the AEX + WL group. Further, appendicular lean mass and VO_2_max were 10% and ~16% lower, respectively, in the WL group than AEX + WL group at baseline (*p*’s < 0.05). T-scores indicated that 39% and 16% of women were osteopenic by femoral neck and total femur regions (no women had osteoporosis at these regions), respectively, while 18% were osteopenic and 6% osteoporotic at the lumbar spine region.

### 3.2. Intervention Effects

Intervention effects may be viewed in Table 1. A group × time interaction was observed for VO_2_max (*p* = 0.03), such that it increased 10% from baseline to 6 months and declined 12% from 6 to 12 months for AEX + WL, with no changes across time observed for WL alone and similar VO_2_max observed between groups by 12 months. There were no group*time interactions or group effects for body weight or BMI, but there was a significant time effect (*p* < 0.01) such that both groups lost ~9% body weight from baseline to 6 months, regained ~2% from 6 to 12 months, with an overall loss of ~7% across the study duration (baseline to 12 months). A time effect was also observed for appendicular lean mass (*p* = 0.02), where groups lost ~3% of lean mass from baseline to 6 months, and continued to lose (~1%) from 6 to 12 months, for an overall loss of ~4% from baseline to 12 months.

No group x time interactions were observed for changes in BMD. However, time effects were observed for total body (*p* < 0.01) and total femur (*p* = 0.03) BMD in that they decreased similar among groups across time (baseline to 6 months: total body: −1.2% and total femur: −1.2%; 6- to 12 months: total body: −0.26% and total femur: −0.09%). No significant effects were observed for femoral neck, Ward’s triangle, greater trochanter, or lumbar spine BMD.

### 3.3. Baseline Predicators of BMD Changes during Weight Regain

Baseline associations may be viewed in Table 2. With regard to the relationship between overall (WL and AEX + WL combine) baseline body composition and changes in BMD during the weight regain phase (6- to 12 months), greater baseline body weight (r = 0.27) and appendicular lean mass (r = 0.35) were associated with less femoral neck BMD loss (*p*’s < 0.05). We also examined the relationship between baseline VO_2_max and changes in BMD during weight regain, observing no significant associations. Further, we examined the relationship between each baseline BMD measure and its change during periods of weight regain. Higher baseline Ward’s triangle BMD was associated with greater declines in Ward’s triangle BMD during weight regain (r = −0.33, *p* < 0.02). No other associations were observed (data not shown).

### 3.4. Weight Loss and Regain Predictors of BMD Changes during Weight Regain

Intervention associations may be viewed in Table 2. WL and AEX groups again were combined for analyses. Greater body weight decline during the WL phase (baseline to 6 months) was associated with less lumbar spine BMD loss during weight regain (r = −0.32, *p* < 0.05), but not any other measure of BMD change during regain. Greater appendicular lean mass decline during the WL phase was associated with greater Ward’s triangle (r = 0.32) and greater trochanter (r = 0.31) BMD loss during weight regain (*p*’s < 0.05). Greater VO_2_max increases during the WL phase were also associated with less BMD loss during the weight regain phase for the total body (r = 0.39, *p* < 0.01), total femur (r = 0.43, *p* < 0.01), Ward’s triangle (r = 0.41, *p* < 0.01), and greater trochanter (r = 0.44, *p* < 0.01), but not femoral neck or lumbar spine. We find that the associations between VO_2_max during the WL phase and BMD loss during the weight regain phase remained present after also adjusting for change in ALM during the WL phase (total body (r = 0.35), total femur (r = 0.39), Ward’s triangle (r = 0.37), and greater trochanter (r = 0.36,)) (*p*’s < 0.05), but again, not for the femoral neck or lumbar spine. Body weight, appendicular lean mass, and VO_2_max changes during the weight regain phase (6- to 12 months) were not associated with any measure of change in BMD during regain. We also examined the relationship between the change in each BMD measure during the WL phase and its change during the weight regain phase. Greater BMD loss during WL is associated with less BMD loss during weight regain for every measure (total body: r = −0.37, *p* < 0.01; femoral neck: r = −0.35, *p* < 0.01; total femur: r = −0.73, *p* < 0.01; Ward’s triangle: r = −0.85, *p* < 0.01; greater trochanter: r = −0.67, *p* < 0.01; lumbar spine: r = −0.34, *p* < 0.04).

## 4. Discussion

This study adds to the body weight management and bone literature by reporting on the 6-month follow-up effects on BMD of WL alone and in combination with AEX in postmenopausal women. Our study supports previous findings [10,11] that the period of weight regain following WL is associated with continued declines in BMD, and adds to the literature by suggesting that this decline occurs regardless of performing AEX during the initial 6 month WL intervention. This is supported by Fogelholm et al. [24] who found that beginning AEX during a 9 month follow up to 3 months of WL did not affect BMD compared to habitual exercise (control) in premenopausal women. However, our findings must be interpreted cautiously because this study may not have been adequately powered to detect changes. In contrast, we also observe weak to moderate positive associations between less loss of appendicular lean mass and greater improvements in VO_2_max during the WL phase and less BMD loss at various anatomical sites during weight regain, suggesting that those who can maintain or improve their muscle quality and functioning during WL, which occurred to a greater extent in the AEX + WL group, may have a greater ability to retain bone longer term.

We observed that ~20% of women had osteopenia and ~10% had osteoporosis at the lumbar spine, which is similar to previous reports in postmenopausal women [6,25]. These data indicate that these women are at moderate risk for future fractures, despite being overweight or obese. The relationship between obesity and osteoporosis has been widely studied, with studies consistently finding that greater body weight is correlated with increased bone mass [26]. However, studies often do not control for the mechanical loading effects of total body weight. A more recent study suggests that after controlling for the mechanical loading effects of body weight on BMD, increased body fat is negatively associated with BMD [27]. It is thought that hormones associated with increased fat mass, such as leptin, may have deleterious effects on bone by affecting bone metabolism, while the increased mechanical muscle force from lean mass during physical activity also may influence bone development.

Beyond lean mass and bone influencing each other through mechanical stress, there are numerous additional mechanisms by which weight-bearing AEX may influence BMD. It is now accepted that muscle and bone interact via paracrine signaling, and likely that factors secreted by contracting muscle (i.e., follistatin, irisin, interleukins) can synergize with mechanical loading to regulate BMD [28]. Further, AEX can affect major autocrine and endocrine pathways (i.e., sex hormones, growth hormone (GH), and insulin-like growth factor-1) that can influence both muscle and bone. Over 600 proteins have been identified as having a potential interaction with bone, including 35 growth factors, 40 cytokines, and 36 metallopeptidases [29]. This is evidenced by muscle contraction via exercise, which induces a highly site-specific mechanical stimulus, affecting distant sites in the skeleton [30], suggesting that muscle affects bone formation via non-mechanical mechanisms. Similar to our findings, previous studies in women support a relationship between maximal aerobic capacity and bone mass and/or quality in the hip [31,32] but not spine [33]. This suggests that in addition to the local effects AEX on muscle, improvements in aerobic capacity also may stimulate systemic changes in several pathways associated with BMD quality (i.e., inflammation, oxidative stress, and insulin signaling) [34]. However, more research is needed to fully understand the influence of aerobic capacity on BMD and why this relationship may be site specific. Further, there is considerable interest in defining the adequate characteristics of exercise (dose, duration, frequency, multimodal intervention) to maximize its mechanical and metabolic effects on BMD, in order to develop appropriate clinical guidelines for bone management.

It must be noted that discrepancies exist in the current literature regarding the effects of weight regain on BMD. Though some studies show that weight regain may partially restore bone loss [10,24], the majority support our findings of minimal or no restoration [5,6,7,8,9,10,11]. Study differences such as mixed genders, younger ages, and study duration make drawing comparisons across studies difficult. These inconsistencies also may be in part due to site-specific BMD responses. We observed that the decline in femoral BMD remained lower at the follow up compared to baseline. This is similar to Kammire et al. [11], who observed that the decline in total hip BMD persisted during a 12 month follow up to an 18 month WL intervention (~3% overall loss), regardless of amount of weight regained. Further, Waters et al. [35] observed that a 30 month follow up after a 1 year WL trial shows that though hip BMD decrease across time, there were no changes in lumbar spine or whole-body BMD. This indicates that the hip region may be especially resistant to bone reformation during periods of weight regain. The hip and spine are identified as two of the most clinically relevant anatomic sites requiring evaluation and potential intervention with aging [36]. There are multiple treatments for loss of BMD, including the use of hormones and dietary modification; however, exercise is the most recommended therapeutic approach [37]. It is suggested that while progressive resistance training (RT) of the lower limbs may be the most effective at improving BMD for the femoral neck, spine BMD may benefit from multimodal interventions, including weight-bearing AEX [38]. When comparing the effects of a 12 month follow up to an 18 month WL alone, AEX + WL, or RT + WL intervention, Beavers et al. [39] observed, after adjusting for total weight change, that BMD in the lumbar spine increased significantly in WL alone and RT + WL, compared to AEX + WL, during the follow up in older adults. This study also found no significant differences between AEX + WL or RT + WL during follow up for total body and hip BMD measurements; however, RT + WL was shown to modestly attenuate total body and femoral neck BMD loss compared to WL alone. Taken together, these data suggest that adding AEX or RT to intentional WL does not prevent BMD loss; however, adding AEX and RT may help to minimize long-term BMD loss.

The results of this study should be interpreted in light of several strengths and limitations. Our measure of objective maximal aerobic capacity via VO_2_max is a strength. The inclusion of only postmenopausal women is also a strength since estrogen is an important regulator of BMD. The lack of a precision study performed on the utilized DXA is a limitation. Precision errors can change over time due to turnover in personnel and wear on the machine, which can diminish the ability to measure true BMD changes [40], particularly with a follow-up duration of only 6 months. The lack of a cellular mechanistic evaluation is also a limitation of the current analyses. Important hormone and pathways regulators to bone (i.e., leptin, cortisol, growth factors, cytokines, and calcium and vitamin D) are needed for evaluation in future studies. Villalon et al. [6] observed that elevations in the bone resorption marker, C-terminal telopeptide of type I collagen, occurring during WL remained elevated during the follow-up period despite weight regain. Because of the small sample size, we were unable to stratify by race; however, our previous study suggests that despite beginning the WL phase with greater BMD than Caucasian women, African American women are not spared from losses of femoral neck and total femur BMD following 6 months of WL [13]. Though this study was not powered a priori to evaluate the short duration of follow-up effects of body weight, lean mass and cardiopulmonary fitness on BMD, our association study results suggest that it is not declines in lean mass or VO_2_max during periods of weight regain, but rather less loss of lean mass and improvements in VO_2_max during weight loss, that may have the greatest influence on longer-term changes in bone.

In summary, results from this analysis support the growing literature suggesting that BMD loss may continue following WL, despite weight regain. Further, this study adds to the literature by suggesting that preserving or improving muscle quality and function may attenuate the loss of BMD during weight regain. Because WL converses multiple health benefits, it is important to identify mechanisms underlying WL-induced bone loss so that effective practices, including exercise, can be designed to minimize the loss of BMD during WL and weight maintenance in older women.

## Figures and Tables

**Table 1:** Participant characteristics at baseline, after 6 months of weight loss with and without aerobic exercise, and after a subsequent 6 month follow up.

		Baseline			6 Months			12 Months			Time	Group	G × T
WL: *N* = 26 and AEX + WL: *N* = 28		Mean	±	SD	Mean	±	SD	Mean	±	SD	*p*-Value	*p*-Value	*p*-Value
Age (yrs)	WL	62.71	±	7.63		-			-		-	-	-
	AEX + WL	58.00	±	6.73		-			-				
Body Weight (kg)	WL	87.65	±	16.90	80.31	±	16.03	81.76	±	16.84	<0.01	0.33	0.77
	AEX + WL	83.87	±	14.17	75.79	±	14.28	77.38	±	15.38			
Body Mass Index (kg/m^2^)	WL	32.91	±	5.86	30.13	±	5.52	30.71	±	6.01	<0.01	0.58	0.68
	AEX + WL	32.20	±	5.68	29.05	±	5.79	29.74	±	6.32			
Appendicular Lean Mass (kg)	WL	20.11	±	2.73	19.18	±	2.32	18.50	±	3.62	0.02	0.09	0.11
	AEX + WL	18.02	±	3.05	17.62	±	2.92	17.81	±	3.08			
VO_2_max (L/min)	WL	1.575	±	0.392	1.426	±	0.255	1.419	±	0.263	0.08	0.07	0.03
	AEX + WL	1.804	±	0.433	1.945	±	0.615	1.649	±	0.347			
Total Body BMD (g/cm^2^)	WL	1.183	±	0.130	1.169	±	0.128	1.165	±	0.128	<0.01	0.97	0.99
	AEX + WL	1.182	±	0.097	1.168	±	0.091	1.165	±	0.094			
Femoral Neck BMD (g/cm^2^)	WL	0.928	±	0.130	0.925	±	0.131	0.921	±	0.140	0.17	0.71	0.56
	AEX + WL	0.913	±	0.108	0.918	±	0.119	0.906	±	0.120			
Total Femur BMD (g/cm^2^)	WL	1.003	±	0.113	0.991	±	0.111	0.984	±	0.111	0.03	0.43	0.70
	AEX + WL	0.975	±	0.112	0.961	±	0.113	0.964	±	0.127			
Ward’s Triangle BMD (g/cm^2^)	WL	0.754	±	0.139	0.738	±	0.148	0.736	±	0.162	0.09	0.84	0.30
	AEX + WL	0.756	±	0.141	0.764	±	0.199	0.735	±	0.137			
Greater Trochanter BMD (g/cm^2^)	WL	0.811	±	0.136	0.801	±	0.137	0.806	±	0.137	0.28	0.39	0.97
	AEX + WL	0.786	±	0.101	0.771	±	0.094	0.779	±	0.100			
Lumbar Spine (L1-L4) BMD (g/cm^2^)	WL	1.174	±	0.122	1.189	±	0.129	1.169	±	0.132	0.07	0.53	0.07
	AEX + WL	1.228	±	0.197	1.211	±	0.200	1.205	±	0.209			

BMD: bone mineral density. WL: weight loss; AEX: aerobic exercise.

**Table 2:** Predictors of BMD changes during weight regain (*N* = 54).

		Total Body BMD (g/cm^2^)	Femoral Neck (g/cm^2^)	Total Femur (g/cm^2^)	Ward’s Triangle (g/cm^2^)	Greater Trochanter (g/cm^2^)	Lumbar Spine (g/cm^2^)
		Change (6–12 Months)	Change (6–12 Months)	Change (6–12 Months)	Change (6–12 Months)	Change (6–12 Months)	Change (6–12 Months)
		r	r	r	r	r	r
Body Weight (kg)	Baseline	0.18	0.27 *	0.07	0.07	0.03	0.20
	Change (0–6 months)	0.26	−0.14	0.10	−0.06	0.04	−0.32 *
	Change (6–12 months)	0.24	0.04	−0.09	0.09	0.00	−0.08
Appendicular Lean Mass (kg)	Baseline	0.16	0.35 *	−0.03	0.17	−0.04	0.08
	Change (0–6 months)	0.18	0.08	0.27	0.32 *	0.31*	0.06
	Change (6–12 months)	0.12	0.17	0.04	−0.07	0.03	0.04
VO_2_max (L/min)	Baseline	−0.03	0.10	0.09	−0.02	0.08	0.29
	Change (0–6 months)	0.39 **	0.04	0.43 **	0.41 **	0.44 **	0.13
	Change (6–12 months)	−0.39	0.12	−0.04	−0.11	−0.05	−0.36

BMD: bone mineral density. Analyses were controlled for age and race; * *p* < 0.05; ** *p* < 0.01.

## Data Availability

The data presented in this study are available on request from the corresponding author.
